# Elimination of Carbides in Carburized Layer of Stainless Steel/Carbon Steel by Horizontal Continuous Liquid–Solid Composite Casting

**DOI:** 10.3390/ma16093516

**Published:** 2023-05-03

**Authors:** Jihong Sun, Xuefeng Liu, Yaohua Yang, Wenjing Wang, Xin Wang, Weiliang Zhang

**Affiliations:** 1 Beijing Advanced Innovation Center for Materials Genome Engineering, University of Science and Technology Beijing, Beijing 100083, China; 2 Beijing Laboratory of Metallic Materials and Processing for Modern Transportation, University of Science and Technology Beijing, Beijing 100083, China; 3 Key Laboratory for Advanced Materials Processing of Ministry of Education, University of Science and Technology Beijing, Beijing 100083, China

**Keywords:** stainless steel/carbon steel clad plates, horizontal continuous liquid–solid composite casting, interfacial carbides, diffusion

## Abstract

The carbides in the carburized layer of stainless steel (SS)/carbon steel (CS) clad plates are prone to inducing intergranular cracks and reducing the interfacial bonding strength. In this paper, SS/CS clad plates were fabricated by horizontal continuous liquid–solid composite casting (HCLSCC), and the formation mechanism of the interfacial carbides and their effect on the elimination of carbides in the carburized layer were revealed by numerical simulation and thermodynamic calculations. During the HCLSCC process, the cladding interface encountered re-melting and re-solidification after rapid melting and solidification, resulting in liquid–liquid and solid–solid diffusion at the cladding interface, where the atomic ratio of Cr/C (Cr/C) gradually increased. Therefore, strip M_7_C_3_ and M_23_C_6_ carbides as well as blocky M_23_C_6_ carbides formed at the cladding interface in turn and had a coherent relationship with the matrix. The blocky M_23_C_6_ carbides led to an increase of 240% in the interfacial ferrite strength. The formation of interfacial carbides reduced the difference in C activity between the cladding interface and SS, thus preventing the diffusion of C to SS and inhibiting carbide precipitation in the carburized layer of SS, which was beneficial to improving the interfacial bonding strength.

## 1. Introduction

The chemical potential difference between SS and CS leads to the uphill diffusion of interfacial C, so that the C is rapidly diffused into the SS [1]. In SS/CS clad plates, a carburized layer forms with carbide precipitation during fabrication by traditional methods, and the carbides in the carburized layer form intergranular cracks and induce corrosion, which decreases the interfacial bonding strength [1,2,3]. In order to avoid the formation of a carburized layer and precipitation of M_23_C_6_ carbides, an interlayer is added to inhibit diffusion of the interfacial elements. However, the strength of the pure metal interlayer is poor [4,5]. The carbides in the carburized layer are dissolved in the matrix after solution treatment, but the heat treatment process is difficult and results in poor SS/CS clad plates [6,7]. When the SS/CS clad plate is subjected to high residual stress, the M_23_C_6_ carbides formed at the grain boundary of the carburized layer lead to reheat cracking and reduce the performance of the clad plate [2,8]. In the high-temperature environment of SS steam reforming tubes, the carbide network coarsens and a precipitation denudation zone forms near the grain boundary, which aggravates grain boundary slip and cracks, resulting in the failure of the reforming tubes. The addition of Nb to SS steam reforming tubes improved the microstructure stability at high temperature, refined the carbide network, inhibited grain boundary slip, and improved the performance of the reforming tubes [9,10]. Therefore, limiting the diffusion of interfacial C during the composite process and inhibiting the precipitation of intergranular carbides in the carburized layer is the key to further improving the comprehensive performance of SS/CS clad plates.

The diffusion of C is determined by its chemical potential, which is mainly affected by the elements Cr and Ni [11]. The strong bond between Cr and C reduces the C diffusion coefficient, decreases the chemical potential of C, and limits the diffusion of C; hence, the chemical potential of C is reduced and the diffusion of C is inhibited by controlling the diffusion of Cr [12,13]. The diffusion of C can control the interfacial carbides in the chromium-rich zone when preparing steel matrix composites by the liquid–solid composite process [14]. During the welding process of SS and CS, the Cr and C atoms mix and diffuse in the weld junction region, forming various types of carbides by changing the value of the Cr/C [15,16]. Hence, constructing laminar interfacial carbides by the liquid–solid composite process, which restricts the diffusion of C and limits carbide precipitation in the carburized layer, is a potential method to improve the interfacial bonding strength.

During the preparation of SS/CS clad plates by liquid–solid composite continuous casting, the CS is prone to coarsening, oxidation, and corrosion at high temperature, and the SS cladding layer easily forms defects such as depressions and cracks. In order to obtain the metallurgical bonding interface by liquid–solid composite continuous casting technology, a higher preheating temperature of the substrate is required. The high temperature leads to the corrosion and oxidation of the substrate, which affects the performance of the clad plate. Liquid–solid composite continuous casting technology is mainly used to prepare composite materials with low melting points or large melting point differences, such as aluminum/aluminum, copper/copper, copper/aluminum, and copper/steel [17,18]. Preparing SS/CS clad plates when the melting points are similar and high is difficult. Only inversion casting and solid–liquid twin-roll casting have been used for the preparation of SS/CS thin strip billet, and the CS was prone to microstructure coarsening, oxidation, and corrosion. The CS near the cladding interface easily formed a ferrite decarburization layer, and the carburized layer of SS easily formed martensite and precipitated carbide, which greatly affected the improvement of the interfacial bonding strength [19,20]. Hence, HCLSCC technology for SS/CS clad plates was developed. A cooling device for carbon steel was designed to prevent microstructure coarsening, excessive oxidation, and corrosion on the surface by controlling the carbon steel temperature. A temperature-controlled inversion solidification device was designed to eliminate defects such as inclusions and pores in the initial inversion solidification layer on the surface of low-temperature carbon steel. At the same time, the device was connected to the water cooling mold to regulate the composite interface structure and the forward/inversion solidification structure of the cladding.

In this paper, 304 SS/Q235 CS clad plates were prepared by HCLSCC. Laminar interfacial carbides formed and the carbides in the carburized layer were eliminated. The formation mechanism of the interfacial carbides and their effect on the elimination of the carbides in the carburized layer were revealed by numerical simulation and thermodynamic calculations.

## 2. Experiment and Mathematical Model Description

### 2.1. Materials and Methods

As shown in Table 1, the chemical composition of 304 SS and Q235 CS was accurately analyzed by spectroscope. The 304 SS was used for the cladding layer (thickness of 4 mm) and the Q235 CS, with a section of 40 mm × 15 mm, was used for the substrate.

The process parameters of the HCLSCC experiment and the simulation were the same. The continuous casting speed (*v*_casting_), the temperature of the SS melt (*T*_ss_), and the temperature of the inversion solidification mold exit (*T*_A_) were changed, respectively. The design of the experiment is shown in Table 2. The preparation of the SS/CS clad plates was realized by multiple process experiments. The tensile and interfacial bonding strengths of the clad plate with eight process parameters were measured, and the with excellent tensile strength and interfacial bonding strength of the clad plate (parameter 8) were selected for analysis.

**Figure 1:**
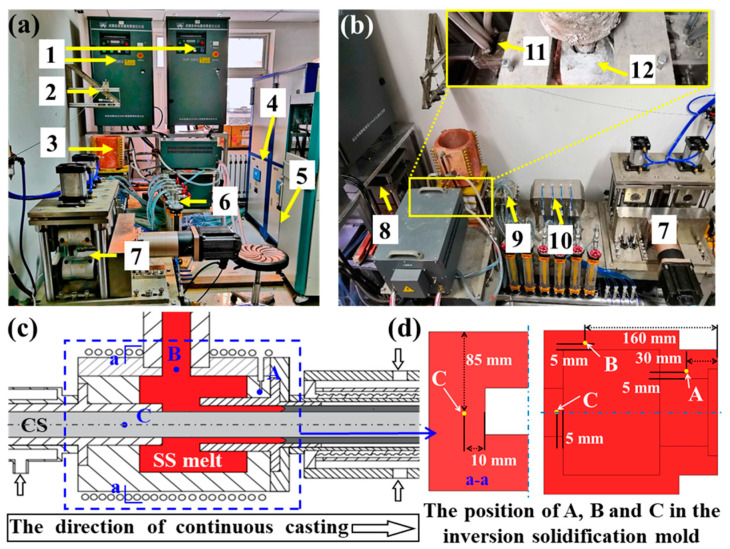
The HCLSCC equipment (**a**,**b**) and sectional drawing of inversion solidification mold (**c**,**d**). (**a**) The main view of the HCLSCC equipment; (**b**) the view of the HCLSCC equipment along the continuous casting direction; (**c**) the plane diagram of the inversion solidification mold; (**d**) the location of temperature measurement points in the inversion solidification mold (In (**c**) and (**d**), A, B and C are the locations of temperature measurement points, and “a” in (C) is section “a-a” in (**d**)). The description of the equipment: 1-heating power; 2-lifting and pulling device; 3-crucible; 4-water chilling unit; 5-control unit; 6-cooling water control; 7-traction device; 8-guided position device; 9-copper cooler; 10-cooling device; 11-substrate plate cooling device; 12-inversion solidification mold.

As shown in Figure 1, the SS/CS clad plates were prepared using the HCLSCC equipment. Firstly, the SS was melted at a heating rate of 0.1 °C·s^−1^, and the temperatures of the inversion solidification mold at positions A, B, and C were 1450, 1550, and 1500 °C, respectively. The cooling water flow rates of the substrate plate cooling device and the copper cooler were 300 and 740 L·h^−1^, respectively. After the SS melt was held at 1550 °C for 30 min, the substrate was moved at a speed of 5 mm·s^−1^ for 50 mm, followed by lifting of the dummy bar. The SS melt flowed into the inversion solidification mold and the copper cooler by a bottom pouring method, contacted the surface of the CS, and then cooled in the air.

**Figure 2:**
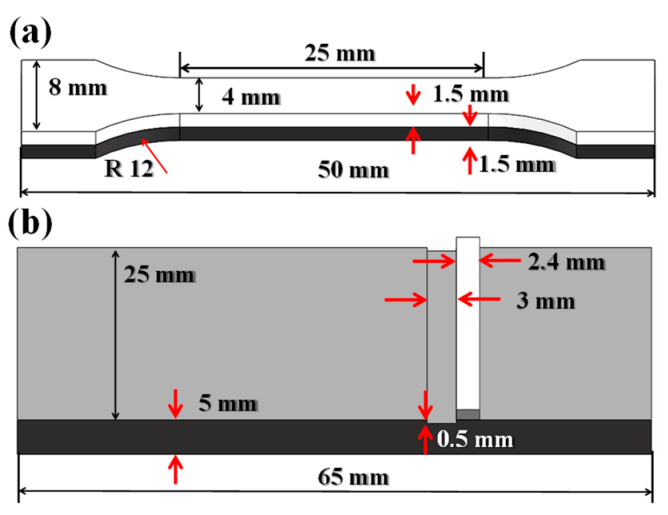
The dimensions of the tensile and shear strength specimens: (**a**) tensile strength specimens; (**b**) shear strength specimens. (The two opposite red arrows represent the size of the distance between them).

The tensile and interfacial shear strengths were tested based on the national standard of China (GB/T 6396-2008 clad steel plates—mechanical and technological test). The dimensions of the specimens are shown in Figure 2. The microstructures of SS and CS were determined using 5 g copper sulfate + 100 mL hydrochloric acid + 100 mL ethyl alcohol and 4 mL HNO_3_ + 100 mL C_2_H_6_O, respectively. The microstructures were observed by scanning electron microscopy using a Gemini SEM 500. The interfacial elements were analyzed by electron probe microanalysis using a JXA-8230. The transmission electron microscopy (TEM) samples were observed using the Titan ETEM G2. The interfacial grain and carbides were measured using ImageJ with five fields of view separately selected for statistical analysis.

### 2.2. Model and Parameters of the Simulation

#### 2.2.1. 3D Model Building and Thermo-Physical Characteristics of Materials

**Figure 3:**
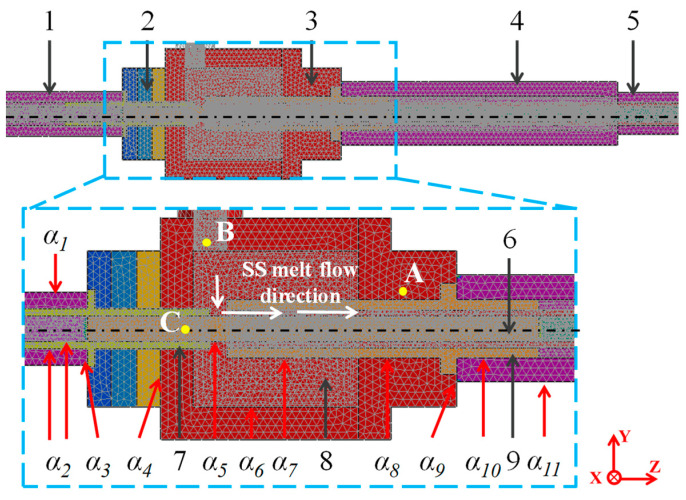
Geometric model of HCLSCC and the location of temperature measurement points(*α_1_*–*α_11_* represents the heat transfer coefficient of the contact surface of each structure of the equipment, and A, B and C are the location of temperature measurement in the HCLSCC process): 1-substrate plate cooling device; 2-heat insulation device; 3-inversion solidification mold; 4-copper cooler; 5-cooling device; 6-CS substrate plate; 7-back connection structure; 8- SS melt; 9-front connection structure.

**Figure 4:**
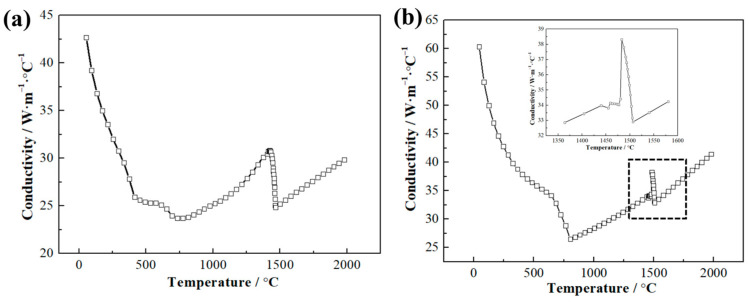
Thermal conductivity of SS (**a**) and CS (**b**).

The geometric model of the HCLSCC process was established to analyze the effect of the continuous casting process on the cladding interface during the composite process. The geometric model of the HCLSCC process and the locations of temperature measurement points A, B, and C are shown in Figure 3. The length of the CS substrate was 1000 mm, and the grid size was 2.5 mm. The length of the SS cladding layer was 760 mm, and the grid size was 1 mm. The grid size of the substrate plate cooling device and the copper cooler was 2.5 mm, and the grid size of other structures was 5 mm. The points A, B, and C were temperature measurement points, and temperature control was performed on point A. Due to the short filling time, the filling process was ignored. The thermo-physical characteristics of the SS and CS were calculated by the simulation software according to the elemental content in Table 1. The thermal conductivity of the SS and CS are shown in Figure 4. The thermo-physical characteristics of the SS and CS are shown in Table 3.

#### 2.2.2. The Mathematical Model

The assumptions of the numerical simulation during the HCLSCC process are below:

(1) SS melt is treated as incompressible Newtonian fluid;
(2) Based on the calculated Reynolds number, SS melt is laminar flow.

The Reynolds number of the 304 SS cladding layer was calculated with a thickness of 4 mm, a maximum SS melt speed of 8.8 mm·s^−1^, a density of 6930 kg·m^−3^, and a dynamic viscosity coefficient of 0.0046 Pa·s (1500 °C). The calculated Reynolds number was 48.9, which was small than 2000 and could be judged as laminar flow.

The main analysis area of the model was the SS melt entering the connection structure and the copper cooler on the surface of the CS. Because it solidified after entering the copper cooler and there were no corners in the front connection structure and the copper cooler, as shown in Figure 3, the entrance position of the SS melt entering the connection structure and the copper cooler was far away from the inner wall of the inversion solidification mold. The corners in the inversion solidification mold had little effect on the SS melt entering the connection structure and the copper cooler, so they were not considered in the present case.

The temperature field control equation used for the HCLSCC process was a non-steady-state heat transfer partial differential equation:

(1) $$\frac{\partial }{\partial x}\left(\lambda \frac{\partial T}{\partial x}\right)+\frac{\partial }{\partial x}\left(\lambda \frac{\partial T}{\partial y}\right)+\frac{\partial }{\partial x}\left(\lambda \frac{\partial T}{\partial z}\right)+\rho L\frac{\partial \beta }{\partial t}=\rho c\frac{\partial T}{\partial t}$$

where *T* is the temperature, °C; λ is the thermal conductivity, W·m^−1^·°C^−1^; *ρ* is the density, kg·m^−3^; *L* is the latent of crystallization released during solidification of the SS melt, J·kg^−1^; *β* is the solid fraction; and *c* is the specific heat of the SS melt, J·kg^−1^·°C^−1^.

The viscosity shear stress per unit area between fluid layers is given by:

(2) $$\iota =\frac{F}{A}=\mu \frac{d\upsilon }{dy}$$

where *ι* is the viscosity shear stress per unit area, Pa; *F* is the friction of the fluid layers, N; *A* is the contact area of the fluid layer, m^2^; *μ* is the dynamic viscosity coefficient, Pa·s; and *υ* is fluid velocity, m·s^−1^.

The Navier–Stokes equation (momentum equation) is given by:

(3) $$\left\{\begin{array}{l} \frac{d\upsilon_{x}}{dt}=f_{x}-\frac{1}{\rho }\cdot \frac{\partial p}{\partial x}+\frac{\mu }{\rho }\left(\frac{\partial^{2}\upsilon_{x}}{\partial x^{2}}+\frac{\partial^{2}\upsilon_{x}}{\partial y^{2}}+\frac{\partial^{2}\upsilon_{x}}{\partial z^{2}}\right)\\ \frac{d\upsilon_{y}}{dt}=f_{y}-\frac{1}{\rho }\cdot \frac{\partial p}{\partial y}+\frac{\mu }{\rho }\left(\frac{\partial^{2}\upsilon_{y}}{\partial x^{2}}+\frac{\partial^{2}\upsilon_{y}}{\partial y^{2}}+\frac{\partial^{2}\upsilon_{y}}{\partial z^{2}}\right)\\ \frac{d\upsilon_{z}}{dt}=f_{z}-\frac{1}{\rho }\cdot \frac{\partial p}{\partial z}+\frac{\mu }{\rho }\left(\frac{\partial^{2}\upsilon_{z}}{\partial x^{2}}+\frac{\partial^{2}\upsilon_{z}}{\partial y^{2}}+\frac{\partial^{2}\upsilon_{z}}{\partial z^{2}}\right) \end{array}\right.$$

where *f* is the external force per unit volume of fluid, and if only gravity is considered, *f* = *ρg*, kg·m^−2^·s^−2^; and *p* is the static pressure, Pa.

The continuity equation is given by:

(4) $$\frac{\partial \left(\rho \upsilon_{x}\right)}{\partial x}+\frac{\partial \left(\rho \upsilon_{y}\right)}{\partial y}+\frac{\partial \left(\rho \upsilon_{z}\right)}{\partial z}+\frac{\partial \rho }{\partial t}=0$$

The energy equation is given by:

(5) $$\left\{\begin{array}{l} \frac{\partial }{\partial t}e_{x}=F_{x}\cdot \upsilon_{x}+\frac{1}{\rho }\cdot \frac{\partial }{\partial x}\left(p_{x}\cdot \upsilon_{x}\right)+\frac{1}{\rho }\left(\lambda \cdot \nabla T\right)+S_{T}\\ \frac{\partial }{\partial t}e_{y}=F_{y}\cdot \upsilon_{y}+\frac{1}{\rho }\cdot \frac{\partial }{\partial y}\left(p_{y}\cdot \upsilon_{y}\right)+\frac{1}{\rho }\left(\lambda \cdot \nabla T\right)+S_{T}\\ \frac{\partial }{\partial t}e_{z}=F_{z}\cdot \upsilon_{z}+\frac{1}{\rho }\cdot \frac{\partial }{\partial z}\left(p_{z}\cdot \upsilon_{z}\right)+\frac{1}{\rho }\left(\lambda \cdot \nabla T\right)+S_{T} \end{array}\right.$$

The *S_T_* of Equation (5) is the contributions of viscous dissipation and latent heat [21]:

(6) $$S_{T}=-L\left(\frac{\partial y}{\partial t}+\nabla \cdot \left(\rho \upsilon \gamma \right)\right)$$

where *γ* is the liquid fraction.

The flow of the SS melt remained laminar. Thus, the boundary layer equation is given by:

(7) $$\left\{\begin{array}{l} \upsilon_{x}\frac{d\upsilon_{x}}{dx}+\upsilon_{y}\frac{d\upsilon_{x}}{dx}=-\frac{1}{\rho }\cdot \frac{\partial p}{\partial x}+\upsilon \frac{\partial^{2}\upsilon_{x}}{\partial y^{2}}\\ \frac{\partial p}{\partial y}=0\\ \frac{d\upsilon_{x}}{dx}+\frac{d\upsilon_{y}}{dy}=0 \end{array}\right.$$

The liquid fraction *γ* of the SS according to the temperature is provided by Equation (8):

(8) $$\gamma =\left\{\begin{array}{c} \begin{array}{l} 0,\;\;\;T_{solidus}>T \end{array}\\ 1,\;\;\;T>T_{liquidus}\\ \frac{T-T_{solidus}}{T_{liquidus}-T_{solidus}},\;\;\;T_{liquidus}>T>T_{solidus} \end{array}\right.$$

where *T_solidus_* is the solid phase line temperature of SS, °C; and *T_liquidus_* is the liquid phase line temperature of SS, °C.

The initial boundary conditions during HCLSCC are shown in Table 4. For the selection of boundary conditions, the reference boundary conditions given in the finite element simulation software and related research on continuous casting were referred [21,22,23,24]. On this basis, the boundary conditions were optimized in comparison to the measured temperatures during the liquid–solid composite continuous casting experiment, which determined the final boundary conditions in Table 4.

## 3. Results

### 3.1. Variations in Temperature Field, Interfacial Heat Flux, and Solid Phase Distribution during the HCLSCC Process

**Figure 5:**
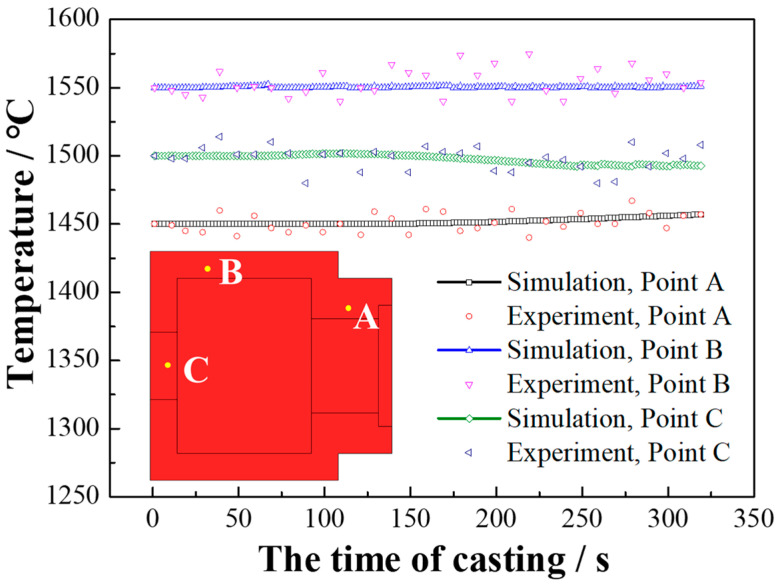
Measured and simulated temperature profiles in the part of the inversion solidification mold at steady state conditions.

Figure 5 displays the results of the experiments conducted according to the process described in Section 2.1 in order to verify the accuracy of the 3D model of the HCLSCC process. Comparing the simulated and measured temperature changes at points A, B, and C, the measured temperatures fluctuated around the simulated temperatures with average errors of 0.42%, 0.52%, and 0.43%, respectively, and maximum errors of 0.96%, 1.61%, and 1.42%, respectively. Therefore, the results of the simulation were in good agreement with the measurements.

**Figure 6:**
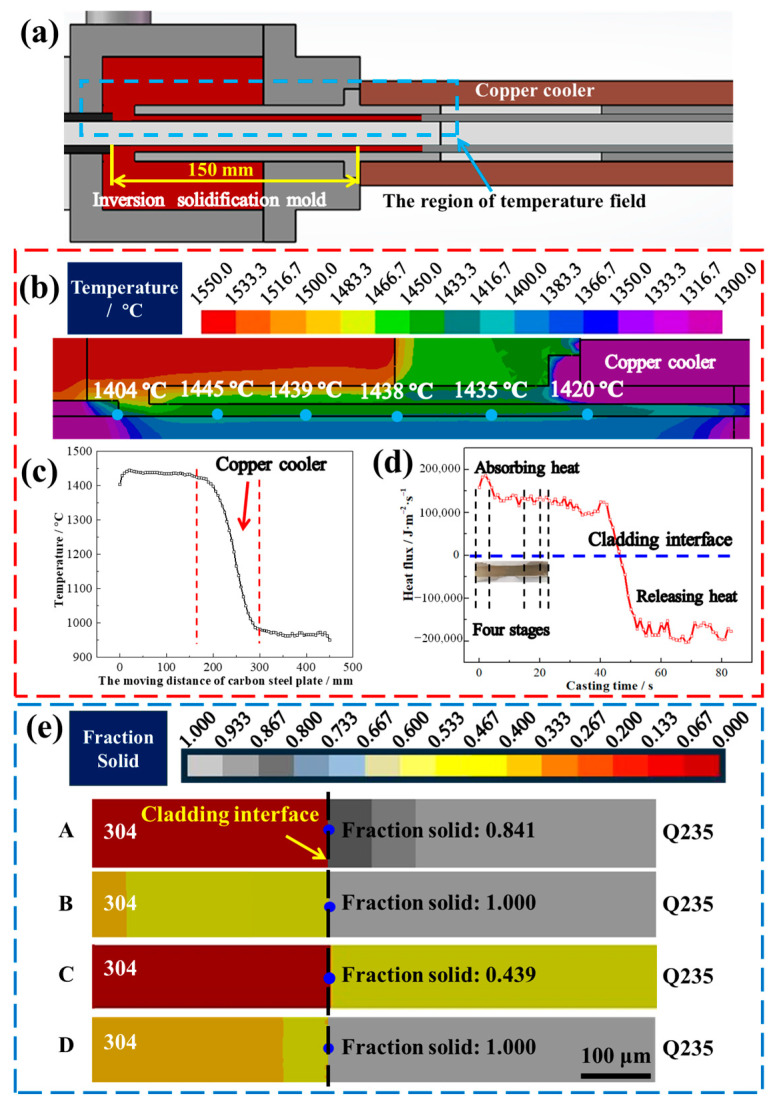
The simulation results during the HCLSCC process: (**a**) the extraction region of the temperature field; (**b**) the temperature field in the inversion solidification mold during the HCLSCC process; (**c**) the change in interfacial temperature; (**d**) the interfacial heat flux; (**e**) the solid phase distribution in the regions A, B, C, and D during the HCLSCC process.

Figure 6 shows the temperature field, interfacial heat flux, and solid phase distribution during the HCLSCC process. As shown in Figure 6a,b, the CS surface temperature rapidly increased after entering the inversion solidification mold, then slowly decreased, and rapidly cooled after entering the copper cooler (Figure 6c). The interfacial heat accumulated gradually during the HCLSCC process (Figure 6d). As shown in Figure 6e, during the HCLSCC process, the CS surface encountered micro-melting (A) and solidification, the minimum solid fraction was 0.841, and the Cr and C mixed. The cladding layer re-melted by the SS melt (B) and the Cr and C continued to diffuse, leading to the width of the carbides increasing (Figure 6e). Then the cladding layer completely re-melted and the CS surface contacted the SS melt and melted (C), with a minimum solid fraction of 0.439. This meant that the proportion of un-melted carbon steel at the cladding interface was 43.9%, the proportion of the SS melt was 56.1%, and the theoretical calculated value of Cr after solidification was 10.60% (considering only the composition of SS and CS mixed).

### 3.2. Microstructure and Element Distribution at the Cladding Interface during the HCLSCC Process

**Figure 7:**
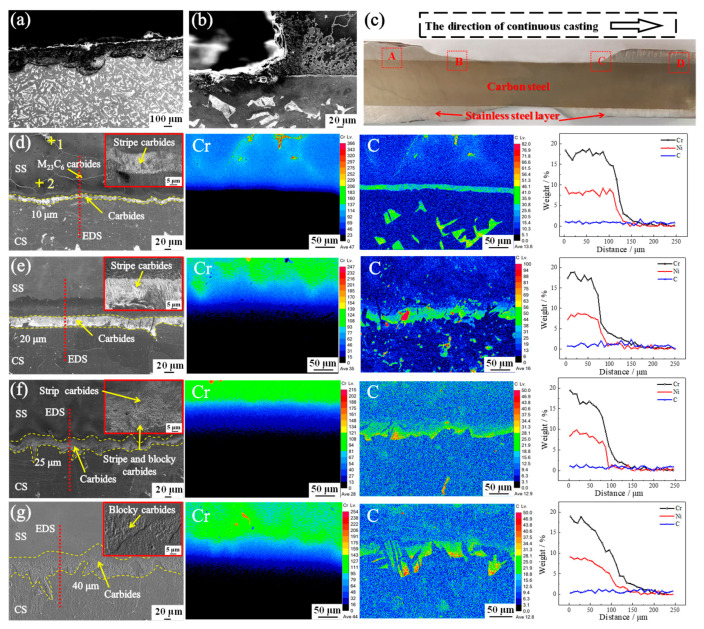
The microstructure and element distribution at the cladding interface: (**a**,**b**) the microstructure of CS; (**c**) the morphology of the cladding layer; (**d**–**g**) the morphology and element distribution of interfacial carbides in the regions A, B, C, and D.

Figure 7 shows the microstructure and element distribution at the cladding interface. The interfacial chemical compositions of regions A, B, C, and D are shown in Table 5. The CS consisted of ferrite and pearlite (Figure 7a), and the CS surface underwent micro-melting during the HCLSCC process, forming a flat cladding interface (Figure 7b). Rapid solidification (A), re-melting (B), re-solidification (C), and complete solidification (D) of the cladding layer occurred with different Cr/C values (Figure 7c). The chemical compositions of the carburized layer of the SS, the cladding interface, and the CS near-interface are shown in Table 5. The interfacial Cr had a gradient distribution and the C was enriched. The interfacial Cr/C was 3:7 (A) and the carbide width was 10 μm (Figure 7d). The Cr and C were enriched in the grain boundary of the carburized layer and led to M_23_C_6_ carbide (4.14C-20.80Cr-75.06Fe wt.%) precipitation in the carburized layer (Figure 7d). As shown in Figure 7e–g, the Cr of the SS in regions B, C, and D occurred with dendrite segregation, the C of the SS in regions B, C, and D had no segregation, and the carbides did not precipitate. The Cr/C values in the regions B, C, and D were 1:1, 3:2, and 3:1, respectively. The width of the carbides increased from 20 to 40 μm and the Cr/C increased. There was no carbide precipitation in the carburized layer of the SS. As shown in Figure 7, the EDS line scan of elements at the cladding interface during the HCLSCC process showed that the Cr and Ni element levels decreased in the CS, and the C element was enriched at the cladding interface. The diffusion distances of the Cr and Ni elements at different stages were 61 and 46 μm, 100 and 85 μm, 129 and 99 μm, and 149 and 116 μm, respectively. The increase in element diffusion distance could improve the alloying degree of the cladding interface.

The diffusion of the Cr element in the liquid–solid composite process was much larger than that in the vacuum welding hot rolling process. In the vacuum welding hot rolling method, the SS and CS were first polished, then welded and vacuumed, and finally rolled at a certain temperature. Although vacuuming can significantly reduce the oxidation of SS and CS during the rolling process and achieve metallurgical bonding, the interfacial oxide cannot be completely eliminated, which still has a certain effect on the interfacial bonding strength. At the same time, due to the difference in chemical potential between the SS and CS, the Cr and Ni in the SS diffused to the CS, and C in the CS diffused to the SS, resulting in the formation of decarburized ferrite in the near-interfacial CS and carbide precipitation in the carburized layer of the SS, which limited the improvement of the interfacial bonding strength and affected the interfacial bonding quality. The diffusion rates of the Cr and C elements in the solid were much smaller than those in the liquid during the HCLSCC process, and the surface temperature of the CS increased rapidly when it contacted the SS melt. By controlling the process parameters such as the temperature of the SS melt, the surface temperature of the CS could be controlled, the temperature of the contact surface between the SS melt and the CS was much higher than the rolling temperature, and the surface of the CS formed a micro-melting zone where the Cr and C elements diffused quickly and mixed. The result was liquid–liquid and solid–solid diffusion at the cladding interface and the atomic ratio of Cr/C increased. After that, a uniformly distributed carbide layer was formed, which could improve the interfacial bonding strength. Thus, the oxide on the surface of the CS was rapidly heated by the SS melt, resulting in high stress and cracks, which then separated from the surface of the CS to achieve the removal of the oxide.

The cladding interface prepared by HCLSCC had no defects such as oxides and pores. During the preparation of SS/CS clad plates, two kinds of defects are mainly formed. One is that inclusions such as oxides are formed at the cladding interface and SS cladding layer, especially in the rapid solidification stage of the cladding layer. The second is that depressions and pores form during the preparation of SS/CS clad plates:

(1) For inclusions such as oxides in the cladding interface and cladding layer. Firstly, before melting, argon was injected into the crucible and the inversion solidification mold to discharge the air in the cavity, and the inversion solidification mold was sealed to prevent air from entering. Secondly, the SS melt was covered during the smelting process to prevent air from entering. At the same time, the crucible adopted the bottom leakage type, and the SS melt flowed from the bottom of the crucible into the inversion solidification mold to reduce mixing of the oxide defects. Finally, during the liquid–solid composite process, the oxide on the surface of CS was removed by the SS melt, and the oxide in the cladding layer was removed by controlling the re-melting of the cladding layer. The removal of the oxide in the cladding interface and layer was realized.
(2) For depressions and pores formed in the cladding layer of the clad plate. Due to the increased temperature of the SS melt, the inversion solidification mold exit, and decreased continuous casting speed, the length of the mushy zone was shortened, which could enhance the feeding capacity of the SS melt and prevent the formation of shrinkage porosity, cracks, and pores during the solidification of the SS melt. The cladding layer of the cladding plate prepared by the HCLSCC process had no defects such as oxides, pores, and cracks, and the microstructure was dense.

## 4. Discussion

### 4.1. The Formation Mechanism of Interfacial Carbides 

**Figure 8:**
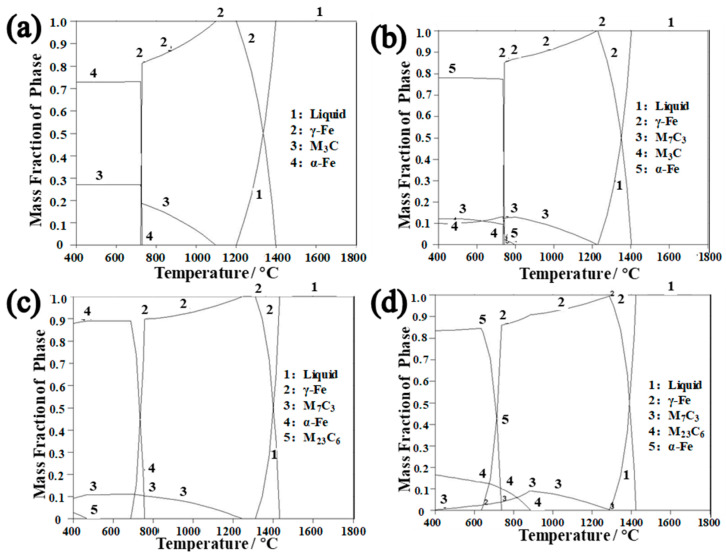
The results of thermodynamic calculations at the regions A, B, C, and D: (**a**) the results of thermodynamic calculations at the A region; (**b**) the results of thermodynamic calculations at the B region; (**c**) the results of thermodynamic calculations at the C region; (**d**) the results of thermodynamic calculations at the D region.

Figure 8 shows the results of the thermodynamic calculations at regions A, B, C, and D, which aligned with the chemical composition of the cladding interface in Table 5. The formation mechanism of the interfacial carbides was analyzed by thermodynamic calculations in Figure 8, revealing that the Cr content of the A region was low, the M_3_C carbides precipitated in the austenite at 1097 °C, and the cladding interface consisted of ferrite and M_3_C carbides (Figure 8a). The Cr content of the B region increased, the M_7_C_3_ carbides precipitated in the austenite at 1223 °C, the cladding interface consisted of ferrite, M_3_C, and M_7_C_3_ carbides at room temperature (Figure 8b), and the width of the carbides increased. The Cr content of the C region increased, the M_7_C_3_ carbides precipitated in the austenite at 1243 °C, the M_7_C_3_→M_23_C_6_ transformation occurred at 469 °C, and a core(M_7_C_3_)–shell(M_23_C_6_) structure was formed (Figure 8c). The Cr content of the D region increased, the M_7_C_3_ carbides precipitated in the austenite at 1289 °C, and the M_7_C_3_→M_23_C_6_ transformation occurred at 886 °C (Figure 8d). The higher interfacial temperature led to the diffusion coefficient increasing and accelerated spheroidization of the carbides. The C gathered laterally in the place where the curvature radius of the carbides was large, so growth of the carbides was promoted laterally (Figure 8d), but they gradually dissolved in the place where the curvature radius was small [25], forming granular and blocky carbides.

### 4.2. Elimination of Carbides in the Carburized Layer of SS 

The carbides in the carburized layer did not precipitate for several reasons. First, the cladding interface re-melted and formed a micro-melting zone, where the Cr and C mixed. The Cr diffused to the cladding interface, which led to the Cr/C increasing, and the formation of higher stability M_23_C_6_ carbides and stabilized interfacial C. Second, the chemical potential difference between the SS and the CS caused the diffusion of C, and C activity played a major role. Based on Equations (9) and (10), the C activity in the carburized layer of the SS, the cladding interface, and the decarburized layer of the CS was calculated when the carbides precipitated [26]:

(9) $$a_{C}=f(\%C)$$

(10) $$\log f_{C}^{\left(Fe-Cr-Ni-C\right)}=\frac{2300}{T}-0.92+\left(\frac{3860+10000y_{Si}}{T}\right)y_{c}+\left(\frac{2000}{T}+0.3\right)y_{Ni}-\left(\frac{9200}{T}-3.05\right)y_{Cr}$$

where *a_C_* is the C activity, *f_C_* is the C activity coefficient, and *y_M_* is the mole fraction of the element. As shown in Table 6, the C activity difference between the cladding interface and the SS decreased from 0.454 to 0.070, the chemical potential gradient of C decreased, and the C diffusion to the SS was weakened, with no obvious diffusion of C (Figure 7g). In addition, the Cr content in the SS gradient decreased, the Cr continuously diffused to the cladding interface, the Cr/C increased, the Cr and C gathered in the CS near-interface, and carbides formed.

Hence, the interfacial carbide hindered the C diffused to the SS, limiting carbide precipitation in the carburized layer of the SS, and formed a coherent relationship with the matrix, thus improving the interfacial bonding strength.

### 4.3. The Strengthening Mechanism of Interfacial Carbides

**Figure 9:**
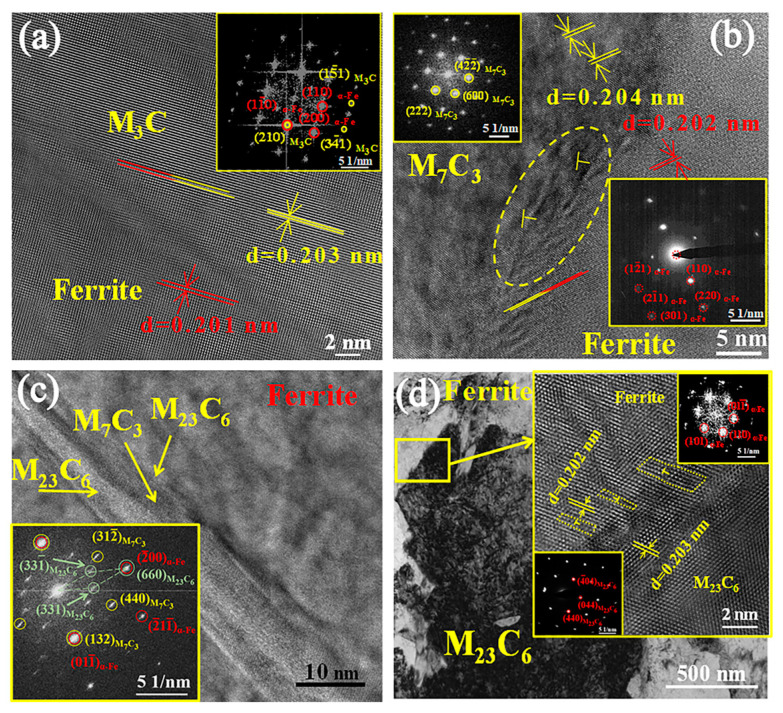
Crystallographic relationships between interfacial carbides and interfacial ferrite matrix in the regions A, B, C, and D: (**a**) the interfacial carbides in the A region; (**b**) the interfacial carbides in the B region; (**c**) the interfacial carbides in the C region; (**d**) the interfacial carbides in the D region.

Figure 9 displays the microstructure of the carbides and relationship between the interfacial carbides and the interfacial ferrite matrix in the regions A, B, C, and D. The crystallographic relationships between the interfacial carbides and the interfacial ferrite matrix in regions A, B, C, and D were [−1211]_M_3_C_∥[00−2]_α-Fe_, (210)_M_3_C_∥(1−10)_α-Fe_, d_M_3_C_ = 0.203 nm, d_α-Fe_ = 0.201 nm (Figure 8a); [01−1]_M_7_C_3__∥[−113]_α-Fe_, (600)_M_7_C_3__∥(110)_α-Fe_, d_M_7_C_3__ = 0.204 nm, d_α-Fe_ = 0.202 nm (Figure 8b); [−11−1]_M_7_C_3__∥[−110]_M_23_C_6__∥[−011]_α-Fe_, (132)_M_7_C_3__∥(01−2)_α-Fe_, (660)_M_7_C_3__∥(−200)_α-Fe_ (Figure 8c); [1−11]_M_23_C_6__∥[−111]_α-Fe_, (440)_M_23_C_6__∥(110)_α-Fe_, (40−4)_M_23_C_6__∥(110)_α-Fe_, d_M_23_C_6__ = 0.203 nm, d_α-Fe_ = 0.202 nm (Figure 8d), respectively. The lattice misfit *δ* of the interfacial carbide and interfacial ferrite matrix could be calculated by Equation (11) [27]:

(11) $$\delta =\frac{2\left|d_{2}-d_{1}\right|}{\left(d_{1}+d_{2}\right)}$$

where *δ* is the lattice misfit, and d_1_ and d_2_ are the crystal plane spacings. In the experiment, *δ_M__3C-(α-Fe)_* = 0.01, *δ_M__7C__3-(__α-Fe)_* = 0.01, and *δ_M__23C__6-(__α-Fe)_* = 0.005 less than 0.05. The interfacial M_3_C, M_7_C_3_, and M_23_C_6_ carbides had a coherent relationship with the matrix.

A coherent relationship between the interfacial carbides and the interfacial ferrite matrix can significantly reduce the coherent stress. At the same time, dislocations between the carbides and the matrix can form a low-density lattice misfit, which reduces the interface energy and improves the interfacial stability and interfacial bonding strength [28,29,30]. The synergistic deformation capacity between the interfacial carbides and the interfacial ferrite matrix could be improved by the precipitation of carbides, leading to the enhanced strength of the matrix. The strengthening effect of the carbides could be evaluated by the Ashby–Orowan equation [31]:

(12) $$\Delta \sigma_{Ashby-Orowan}=\frac{0.538Gbf^{\frac{1}{2}}}{d}\ln\frac{d}{2b}$$

where Δ*σ_Ashby-Orowan_* is the increment of precipitation strengthening, *G* is the shear modulus [32], *b* is the burgers vector [32], *f* is the volume fraction (*f* = 11.7 vol.%), and *d* is the average diameter of the precipitates (*d* = 62.5 nm). Based on Equation (12), Δ*σ_Ashby-Orowan_* = 288 MPa, the theoretical calculated value of interfacial bonding strength was 408 MPa (only the precipitation strengthening of carbides was considered). The blocky M_23_C_6_ carbides led to an increase of 240% in the interfacial ferrite strength (*σ_ferrite_* = 120 MPa [33]).

**Figure 10:**
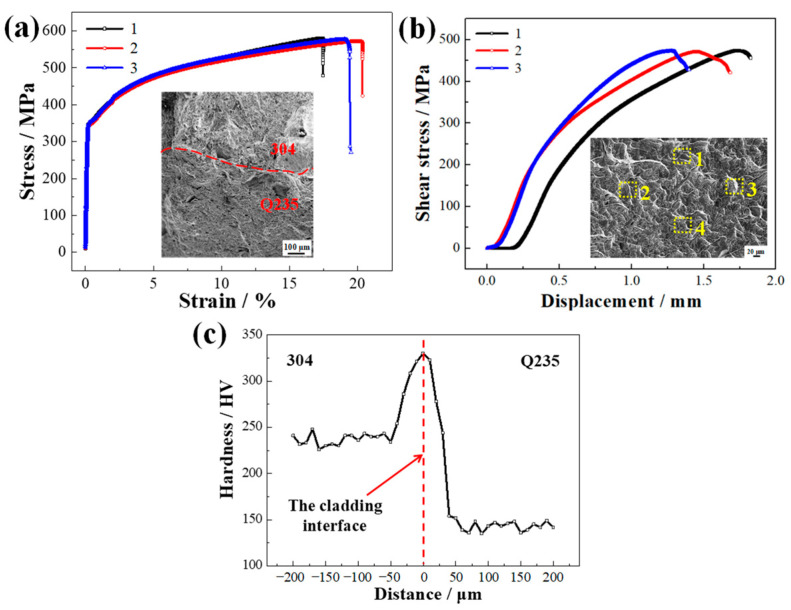
The mechanical properties of the SS/CS clad plates: (**a**) the stress–strain curves; (**b**) the tensile shear stress–displacement curves; (**c**) the micro-hardness near the cladding interface of the SS/CS clad plates. (The 1–4 is the scanning area of EDS in (**b**)).

Figure 10 displays the tensile strength, interfacial shear strength, and micro-hardness near the cladding interface of the SS/CS clad plates with blocky M_23_C_6_ carbides formed at the cladding interface. Curves 1, 2, and 3 in Figure 10a are the stress–strain curves of different specimens under the same process. Curves 1, 2, and 3 in Figure 10b are the shear strain–displacement curves of different specimens under the same process. As shown in Figure 10a, the yield and tensile strengths were 381 and 577 MPa, respectively. As shown in Figure 10b, the interfacial tensile shear strength was 473 MPa, and the regions of 1-4 is the scanning area of EDS in (b). The interfacial tensile shear strength of the SS/CS clad plates prepared by vacuum welding hot rolling with a rolling temperature of 1150 °C and rolling reduction of 80% was 389 MPa [2]. The cladding interface tensile shear strength of the clad plates prepared by HCLSCC was similar to the clad plates prepared by vacuum welding hot rolling. Combined with the fracture morphology, the tensile fracture was ductile with a large number of dimples, and the cladding and substrate layers were not delaminated, which further illustrated that limiting the precipitation of carbides in the carburized layer could limit the formation of cracks and improve the strength of the clad plate. At the same time, according to the compression–shear fracture morphology, the fracture occurred on the CS near-interface, with large numbers of tearing edges and dimples, which significantly improved the interfacial bonding strength. Figure 10c shows the hardness distribution of the cladding interface. The maximum hardness at the cladding interface was 330 HV. The hardness gradually decreased from this point to the SS and CS. The average hardness values of the SS and CS were 237 HV and 143 HV, respectively.

**Figure 11:**
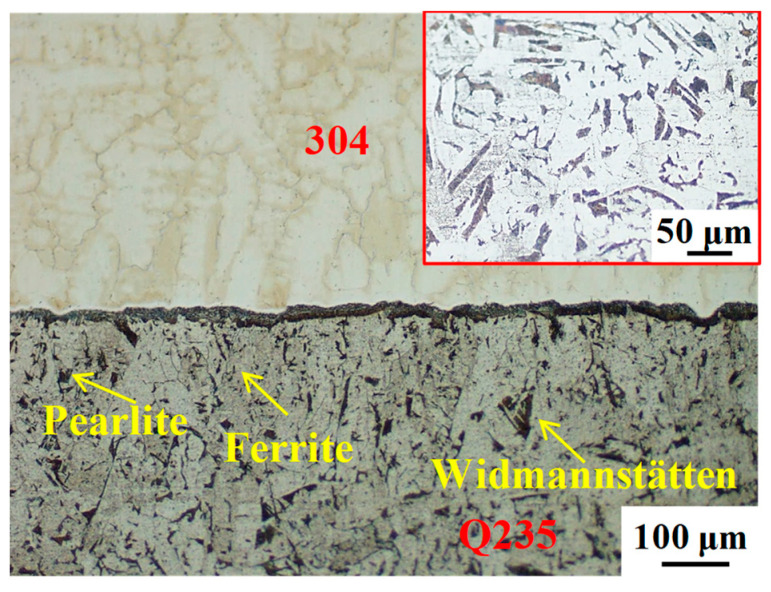
Microstructure of the cladding interface.

Blocky carbides formed in steel or during the welding process can preferentially act as crack initiation centers and cause fractures. For SS/CS clad plates prepared by hot rolling or diffusion bonding, stress concentration occurs in the low-strength ferrite decarburization layer under stress conditions, where the crack will preferentially initiate, propagate, and finally fracture. As shown in Figure 11, the interface region of the clad plate prepared by HCLSCC was composed of austenite on the SS, interfacial carbides, and widmannstätten, pearlite, and ferrite on the CS. For the SS/CS clad plate prepared by HCLSCC, the blocky carbides were constructed on the ferrite matrix near the cladding interface and eliminated in the ferrite decarburization layer, which strengthened the ferrite and improved the synergistic deformation ability of the clad plate. Under stress conditions, synchronous deformation of the interface reduced stress concentration at the interfacial carbides and delayed the formation of cracks. At the same time, the uniform distribution of carbides at the cladding interface can significantly alleviate the formation of cracks [34,35]. Compared with the blocky carbides formed at the cladding interface, the carbides in the widmannstätten of the CS were lamellar and provided a relatively easy path for the formation and propagation of cracks, making it easier to form cracks and resulting in fracture of the clad plate. Table 7 shows the element content in the regions 1–4 of the shear fracture in Figure 10b. The fracture surface contained a large amount of Fe and almost no Cr and Ni, indicating that the clad plate fractured at the CS due to the formation of cracks in the widmannstätten of the CS. Therefore, the interfacial blocky carbides reduced the probability of crack formation at the cladding interface of SS/CS clad plate prepared by HCLSCC. Additionally, the stress concentration caused by carbides was eliminated and the sensitivity of crack formation was reduced due to no lamellar carbides precipitating in the PFZ of the SS near-interface [36]. Therefore, the interface bonding strength of the composite plate prepared by HCLSCC was significantly improved, and the cracking position was located in the widmannstätten zone far from the interface. The volume change caused by solidification shrinkage and phase transformation will lead to the formation of residual stress at the interface due to the different thermo-physical parameters of austenite and ferrite and uneven temperature distribution during HCLSCC [22,37].

The cladding interface formed the blocky M_23_C_6_ carbides was coherent with the matrix, which significantly enhanced the interfacial bonding strength. At the same time, the improvement of the interfacial bonding strength strengthened the clad plate. There was no decarburization layer on the CS near the cladding interface, and no carbides were formed on the carburized layer of the SS. There were no defects such as oxides and pores at the cladding interface, which further enhanced the strength of the clad plate.

Hence, high-quality SS/CS clad plates could be prepared by the HCLSCC process, which may introduce a promising method to integrate control of the microstructure and the performance of laminated composites.

## 5. Conclusions

The cladding layer underwent rapid solidification, re-melting, re-solidification, and forward/inversion solidification to form a dense and defect-free cladding layer. The surface of the CS underwent rapid solidification and re-melting of the cladding layer and formed a micro-melting zone where the gradient of the liquid phase volume fraction was small, and the Cr and C elements were uniformly mixed as the surface of the CS contacted the SS melt. The result was liquid–liquid and solid–solid diffusion at the cladding interface, achieving metallurgical bonding and gradually increasing the Cr/C.

The formation of a uniformly distributed blocky M_23_C_6_ carbide layer at the cladding interface reduced the C activity difference between the cladding interface and the SS near-interface from 0.454 to 0.070. Reducing the chemical potential gradient of C and hindering the diffusion of C to the SS inhibited the precipitation of carbides in the carburized layer, thus eliminating the decarburization layer. 

The tensile and interfacial bonding strengths of the SS/CS clad plates were 577 and 473 MPa, respectively. The interfacial Cr/C gradually increased, leading to the formation of strip M_7_C_3_ and M_23_C_6_ carbides as well as blocky M_23_C_6_ carbides in turn, which had a coherent relationship with the matrix. The blocky M_23_C_6_ carbides at the cladding interface contributed to a 288 MPa increase in ferrite strength, corresponding to an increase of 240%. At the same time, the improvement of the interfacial bonding strength strengthened the clad plates. There was no decarburization layer on the CS near the cladding interface, and no carbides were formed on the carburized layer of the SS. There were no defects such as oxides and pores at the cladding interface, which further enhanced the strength of the clad plate.

## Figures and Tables

**Table 1:** The chemical composition of cladding layer and substrate (wt.%).

	C	P	Mn	Si	S	Cr	Ni	Fe
SS cladding layer	0.07	0.04	1.218	0.09	0.02	18.87	9.06	Bal.
Standard of S30408	0.08	0.045	2.00	1.00	0.030	18.00–20.00	8.00–11.00	
CS substrate	0.178	0.045	1.885	0.03	0.05	--	--	Bal.
Standard of Q235	≤0.22	≤0.045	≤1.40	≤0.035	≤0.050	--	--	--

**Table 2:** The parameters of the experiment and the simulation.

Parameters	*T_ss_*/°C	*T_A_*/°C	*v_casting_*/mm·s^−1^
1	1550	1250	1
2	1550	1250	2
3	1550	1250	5
4	1550	1250	10
5	1500	1350	5
6	1530	1350	5
7	1550	1350	5
8	1550	1450	5

**Table 3:** The solid/liquid phase line temperature and the specific heat capacity of SS and CS.

	Quantity	Unit	Value
*T_s-304_*	Solid phase line temperature of SS	°C	1403
*T_l-304_*	Liquid phase line temperature of SS	°C	1463
*T_s-Q235_*	Solid phase line temperature of CS	°C	1430
*T_l-Q235_*	Liquid phase line temperature of CS	°C	1504
*C_ss_*	Specific heat capacity of SS	kJ·kg^−1^·°C^−1^	0.50
*C_cs_*	Specific heat capacity of CS	kJ·kg^−1^·°C^−1^	0.46

**Table 4:** The boundary conditions and heat transfer coefficient (HTC) for HCLSCC.

	Quantity	Unit	Value
*T_ss_*	Temperature of SS melt	°C	1550
*T_cs_*	Temperature of CS	°C	1000
*v_cast_*	Continuous casting speed	mm·s^−1^	5
*T*_1_	Temperature of the inversion solidification mold	°C	1550
*T*_2_	Temperature of the inversion solidification mold exit	°C	1450
*α_1_*	HTC between copper wall of substrate plate cooling device and cooling water	W·m^−2^·°C^−1^	5000
*α_2_*	HTC between CS and back connection structure with the gap	W·m^−2^·°C^−1^	10
*α_3_*	HTC between substrate plate cooling device and heat insulation device	W·m^−2^·°C^−1^	100
*α_4_*	HTC between heat insulation device and inversion solidification mold	W·m^−2^·°C^−1^	100
*α_5_*	HTC between SS melt and CS	W·m^−2^·°C^−1^	780,000
*α_6_*	HTC between SS melt and inversion solidification mold	W·m^−2^·°C^−1^	3000
*α_7_*	HTC between SS melt and front connection structure	W·m^−2^·°C^−1^	5000
*α_8_*	HTC between front connection structure and inversion solidification mold	W·m^−2^·°C^−1^	3000
*α_9_*	HTC between copper cooler and inversion solidification mold with heat insulation device	W·m^−2^·°C^−1^	10
*α_10_*	HTC between copper cooler and front connection structure	W·m^−2^·°C^−1^	3000
*α_11_*	HTC between copper wall of copper cooler and cooling water	W·m^−2^·°C^−1^	5000

**Table 5:** The chemical composition of the cladding interface in the regions A, B, C, and D (wt.%).

Region	Location	Cr	Ni	C	Si	Fe
A	Carburized layer of SS	11.21	2.73	0.73	0.04	Bal.
	interface	3.28	0.33	1.22	0.03	Bal.
	CS near-interface	1.03	0.22	0.34	0.02	Bal.
B	Carburized layer of SS	10.77	7.35	0.88	0.04	Bal.
	interface	6.33	1.47	1.72	0.03	Bal.
	CS near-interface	1.39	1.10	0.13	0.02	Bal.
C	Carburized layer of SS	15.83	8.11	0.41	0.04	Bal.
	interface	12.44	2.44	0.98	0.03	Bal.
	CS near-interface	2.11	1.76	0.15	0.02	Bal.
D	Carburized layer of SS	17.71	8.57	0.48	0.04	Bal.
	interface	15.53	3.11	0.94	0.03	Bal.
	CS near-interface	2.77	2.07	0.17	0.02	Bal.

**Table 6:** The C activity in the regions A, B, C, and D.

Region	Carburized Layer of SS	Cladding Interface	CS Near-Interface
A	0.724	1.178	1.184
B	0.722	0.923	0.932
C	0.363	0.552	0.884
D	0.318	0.388	0.803

**Table 7:** The element content in the regions 1–4 of the shear fracture (wt.%).

Region	Cr	Ni	C	Fe
1	0.8	0.2	1.4	Bal.
2	0.6	0.3	1.0	Bal.
3	0.5	0.1	1.5	Bal.
4	1.5	0.1	1.0	Bal.

## Data Availability

The data that support the findings of this study are available from the corresponding author, [X.L.], upon reasonable request.
